# Faecal metagenomes of great tits and blue tits provide insights into host, diet, pathogens and microbial biodiversity

**DOI:** 10.1099/acmi.0.000910.v3

**Published:** 2025-04-28

**Authors:** Mark J. Pallen, Alise Jany Ponsero, Andrea Telatin, Cara-Jane Moss, David Baker, Darren Heavens, Gabrielle L. Davidson

**Affiliations:** 1 Quadram Institute Bioscience, Norwich Research Park, Norwich, UK; 2 University of East Anglia, Norwich Research Park, Norwich, UK; 3 Earlham Institute, Norwich Research Park, Norwich, Norfolk, NR4 7UZ UK; 4 University of Cambridge, Downing Street, Cambridge, CB2 3EB, UK

**Keywords:** antimicrobial resistance, *Cyanistes caeruleus*, FONA beta-lactamase, gut microbiome, *Isospora*, metagenomics, *Parus major*, *Serratia fonticola*, siadenovirus

## Abstract

**Background.** The vertebrate gut microbiome plays crucial roles in host health and disease. However, there is limited information on the microbiomes of wild birds, most of which is restricted to barcode sequences. We therefore explored the use of shotgun metagenomics on the faecal microbiomes of two wild bird species widely used as model organisms in ecological studies: the great tit (*Parus major*) and the Eurasian blue tit (*Cyanistes caeruleus*).

**Results.** Short-read sequencing of five faecal samples generated a metagenomic dataset, revealing substantial variation in composition between samples. Reference-based profiling with Kraken2 identified key differences in the ratios of reads assigned to host, diet and microbes. Some samples showed high abundance of potential pathogens, including siadenoviruses, coccidian parasites and the antimicrobial-resistant bacterial species *Serratia fonticola*. From metagenome assemblies, we obtained complete mitochondrial genomes from the host species and from *Isospora* spp., while metagenome-assembled genomes documented new prokaryotic species.

**Conclusions.** Here, we have shown the utility of shotgun metagenomics in uncovering microbial diversity beyond what is possible with 16S rRNA gene sequencing. These findings provide a foundation for future hypothesis testing and microbiome manipulation to improve fitness in wild bird populations. The study also highlights the potential role of wild birds in the dissemination of antimicrobial resistance.

**Impact Statement**

The community of micro-organisms that lives in an animal’s gut is crucial for the host’s health. However, in wild birds, such as the great tit and Eurasian blue tit, the role of this community is poorly understood. Here, we have applied advanced DNA sequencing and computational techniques to faecal samples from these birds, revealing a rich diversity of microbes, including previously unknown species and potential pathogens. We discovered antibiotic resistance genes, raising concerns about the potential spread of resistance from wild birds to humans. This research highlights the importance of understanding gut microbes in wild birds and lays the groundwork for future efforts to manipulate their gut microbial communities to enhance their health and resilience in changing environments.

## Data Summary

The authors confirm that all supporting data, code and protocols have been provided within the article or through supplementary data files. Metagenome sequences are available in the International Nucleotide Sequence Database Collaboration under BioProject ID PRJEB79635, with accession numbers ERX13007473, ERX13007474, ERX13007475, ERX13007476 and ERX13007477. We have made the MAG catalogue available in Zenodo archive (https://doi.org/10.5281/zenodo.13643296), the viral sequence catalogue available at https://doi.org/10.5281/zenodo.13643307 and the mitochondrial sequences available at https://doi.org/10.5281/zenodo.13734416. Supplementary tables are available from https://doi.org/10.6084/m9.figshare.26968957 [1].

## Introduction

The vertebrate gut microbiome plays crucial roles in animal health and host fitness, including nutrient recovery, protection from infection and priming normal development of the gut and immune system [2,3]. Additionally, this key microbial community can act as a reservoir of pathogens and antimicrobial resistance [4,5], as well as a source of biotechnological resources [6]. As a result, the gut microbiome has been a major focus of intensive research in humans and domesticated animals [7,9]. Variation in the gut microbiome is also likely to affect host health fitness in wild animals, including birds, although few experimental studies have tested this hypothesis [10,11].

Wild birds face numerous threats associated with the Anthropocene, including climate change, habitat loss and reduction in genetic diversity [12]. Endangered populations, whether in the wild or in captivity, are also likely to experience a loss of microbial diversity in the gut, which can negatively impact fitness [13,14]. In addition, variation in parasite load, interacting with the gut microbiome, is likely to affect survival [15,18]. In these circumstances, microbiome manipulation offers a potential path to enhance fitness for individual birds and for endangered populations [19]. Understanding how the gut microbiome impacts wildlife hosts is an important goal for conservation and evolutionary biology.

The great tit (*Parus major*) and the Eurasian blue tit (*Cyanistes caeruleus*) are small passerines from the family *Paridae*, widely used as model species in ecological studies [20,22]. These species are experimentally tractable because they are altricial (growing quickly, while remaining in their nests until near independence) and so are easily monitored in nest boxes. They are also widespread and common species across Eurasia, where they have adapted to human-modified habitats, dominating garden bird feeders. They also represent potentially important vectors of zoonotic pathogens, such as *Campylobacter* and *Borrelia* [23,24]. Although not endangered, these species have experienced unusual mortality incidents in recent decades in the UK and Germany, linked to bacterial and viral pathogens [25,27]. Consequently, they provide an attractive test case for investigating microbial biodiversity within the gut microbiomes of wild birds.

Previous studies of parid gut microbiomes have documented a rich diversity of micro-organisms and variation in taxonomic composition based on age and diet [28,33]. However, these efforts have largely relied on metabarcoding with incomplete 16S rRNA gene sequences. Unfortunately, this approach fails to provide species-level resolution, cannot detect viruses or eukaryotes and provides no insights into the genome sequences, population structures or functional repertoires of resident microbial species [34]. Thus, despite previous efforts, the great tit and blue tit gut microbiomes present us with a largely unexplored landscape of taxonomic, ecological and functional diversity, with potentially important, undiscovered roles in fitness, while also encompassing known and newly discovered parasites.

The application of shotgun metagenomics to gut microbiomes has proven capable of linking host, diet and microbes, while also documenting microbial diversity through reference-based phylogenetic profiling and recovery of genome sequences through assembly and binning [9,35,38]. Here, we apply shotgun metagenomics to five parid faecal samples, providing proof of principle for sampling, sequencing and bioinformatic methods, while also laying the foundations for hypothesis testing and microbiome manipulation in wild birds.

## Methods

### Sample collection and analysis

This study was conducted with ethical approval from the University of East Anglia Animal Welfare and Ethical Review Body (AWERB) (Application ID: ETH2223-2022) and the University of Cambridge AWERB (Application ID: Z0067/19 and Z0068/19). Faecal samples (Table S1, available in the online Supplementary Material) were collected from

- nest boxes in Madingley Wood, Cambridge, UK, from one full-grown great tit in October 2019 and from five great tit nestlings in May–June 2020. Nests were monitored for breeding progress, and samples were collected in sterile bags [39] from the adult and from nestlings when chicks were 15 days old, following our established protocol [28].
- six full-grown blue tits, using mist nets at the University of East Anglia, Norwich, UK, in November 2023.

Samples from great tits were stored in 100% ethanol, while those from blue tits were stored in 1:1 DNA/RNA Shield Solution (Zymo Research). Samples were stored at −80 °C within a few hours of collection and until DNA extraction. DNA was extracted using the QIAamp PowerFecal Pro DNA Kit (Qiagen; 51804) following the supplier’s protocol, with modifications to increase DNA yield.

During DNA extractions on the great tit samples performed in 2021, the bead tube was vortexed for 10 min and samples were heated in a heat block at 65 °C for 15 min and this step was repeated three times. The CD2 solution step was repeated to remove any potential inhibitors, and samples were washed with C5 twice. DNA was eluted in 50 µl 10 mM Tris-HCI and stored at −80 °C until further analysis.

During DNA extractions on the blue tit samples performed in 2023, samples were homogenized with zirconium beads in CD1 solution and beaten in the portable homogenizer SuperFast Prep-2 (MP Biomedicals, USA) for 25 s at speed code 20. The supernatant was recovered and subjected to 1× bead clean-up using Kapa pure beads (Roche Catalogue No. 07983298001), followed by two 70% ethanol washes, and DNA was eluted in 20 µl 10 mM Tris-HCl. Extracted DNA was stored at −80 °C before further analysis.

### Metagenomic sequencing

DNA quantification was performed using a Qubit™ 3.0 fluorometer (Invitrogen, CA) and dsDNA HS assay kit. Samples were concentrated through an AMPure bead clean-up, using a 1.5× bead volume. DNA was resuspended in 6 µl PCR grade water, the full volume of which was used in the tagmentation reaction of library preparation.

Illumina sequencing libraries were constructed using the Nextera XT library preparation kit according to the manufacturer’s recommendations.

The quality of the libraries was assessed using the Agilent 2200 TapeStation system. Seven samples that failed to yield DNA libraries of sufficient quality for sequencing were excluded from further analysis, leaving (Table 1):

- two samples from the great tit *P. major* (TV34554 and TV35040) from 15-day-old fledglings

**Table 1.** Subject and sample characteristics

Bird ID	Collection date	Site code	Site	Coordinate	Species	Age	Sex	Capture method
ABR6116	11 November 2023	UEARE	UEA Norwich	52.616424, 1.234345	Blue tit	Juvenile	Female	Mist net
ABR6151	11 November 2023	UEARE	UEA Norwich	52.616424, 1.234345	Blue tit	Adult	Male	Mist net
ABR6154	11 November 2023	UEARE	UEA Norwich	52.616424, 1.234345	Blue tit	Juvenile	Unknown	Mist net
TV34554	16 May 2020	MW73	Madingley Cambridge	52.21788, 0.04674	Great tit	15 days	Unknown	Nest box
TV35040	25 May 2020	MW89	Madingley Cambridge	52.2171, 0.04985	Great tit	15 days	Unknown	Nest box

- three samples from the blue tit, *Cyanistes caeruleus* (one adult ABR6151 and two juveniles, ABR6116 and ABR6154).

Successful libraries were equimolar pooled, and the final pool was double size selected by solid phase reversibile immobilisation between 0.5× and 0.7× bead volumes using AMPure beads. The final pool was quantified using a Qubit™ 3.0 fluorometer (Invitrogen) and run on the Agilent 2200 TapeStation system to calculate the molarity.

Paired-end metagenomic sequencing was performed on the Illumina NextSeq 2000 platform at the Quadram Institute Norwich, yielding 2×150 bp paired-end sequencing reads.

### Metagenomic analysis

Metagenomic reads were trimmed and quality controlled using FastP configured to a minimum phred score of 20 and minimum length of 50 bp [40]. Taxonomic profiling of sequencing reads was performed using Kraken2 v2.1.0 [41], with a microbial database built from archaeal, bacterial, fungal, protozoan, viral and univec_core sequences in RefSeq in 2024.

Individual assemblies were performed on each metagenomic sample using MEGAHIT v1.2.9 [42]. The blast 2.16 suite of programmes [43] was downloaded from ftp.ncbi.nlm.nih.gov/blast and used to perform homology searches of the assemblies, using the *makeblastdb* utility to generate libraries, the *blastn* and tblastx utilities to search contigs with query sequences under high stringency (*e* value ≥1*e-200) and the *blastdbcmd* utility to retrieve hits from databases.

Antibiotic and metal resistance genes were identified from the assembled contigs using Abricate v1.0.1 (https://github.com/tseemann/abricate) against the PanRes database v1.0.1 (available at https://zenodo.org/records/8055116). The potential host of these ARGs (antibiotic resistance genes) was inferred by taxonomic classification of the contigs carrying ARGs using Kraken2 against the RefSeq database.

To avoid contamination of the bins by eukaryotic sequences, Tiara v1.0.3 [44] was used to classify contigs longer than 3,000 kb into their high-level kingdoms, allowing to exclude sequences of a eukaryotic or of an organelle origin and only retaining all unclassified contigs and prokaryotic contigs for the binning step.

Contigs were binned using MaxBin2 v2.2.7, SemiBin2 v2.1.0 and Metabat2 v2.15 independently [45,47]. The bins were refined using DasTool v1.1.7 [48] using a min score threshold of 0.3. The quality of the refined bins was obtained using CheckM2 v1.0.2 [49], and any bins with contamination above 10% were excluded. The final metagenome-assembled genomes (MAGs) were classified as low quality (<50% completeness and <10% contamination), medium quality (>50% completeness and <10% contamination) and high quality (>90% completeness and <5% contamination), as recommended by the MIMAG specification [50]. Finally, the MAGs were dereplicated using dRep v3.4.3 [51] with an average nt identity (ANI) of 95% and classified using gtdb-tk v2.4.0 on the Genome Taxonomy Database (GTDB) Release 220 [52,54].

DRAM v1.5 [55] was run against the DRAM database downloaded on July 2024 on the MAGs. Briefly, DRAM first annotates the genes of each MAG using the UniRef90 [56], Pfam [57] and dbCan [58] databases. Then, a secondary step (distillation) is run to curate these annotations into useful functional categories, allowing to infer the main metabolic functions detected in the MAG.

The potential viral and plasmid sequences were retrieved from the assembled contigs using GeNomad v1.8.0 [59], and the quality of these viral sequences was then assessed using CheckV v1.0.3 [60] using the CheckV database v1.5. Putative viral sequences longer than 1,000 bp carrying at least one hallmark viral gene or no detectable cellular genes were considered in this analysis. The viral sequences were clustered into species-level viral operational taxonomic unit (vOTU) clusters using mmseqs2 v2.13 [61] using an identity threshold of 95% over 75% of the longest sequence. For viral species identified as belonging to the Caudovirales, the potential host was inferred using iPhop v1.3.3 [62].

To estimate the relative abundance of the vOTUs in the samples, Bowtie2 v2.5.4 [63] was used to map the short reads after quality control to the vOTU representative sequences, generating a count table that represents the number of reads mapped to each contig in the original sample. Finally, bbmap v39.01 (https://jgi.doe.gov/data-and-tools/software-tools/bbtools/) was used to normalize the read counts in read counts per kb million reads, corresponding to the read counts normalized by the vOTU reference sequence length in kb and by the total of reads mapped to the viral catalogue.

## Results and discussion

### Reference-based profiling provides insights into host diet, gut microbiota and pathogens

Short-read sequencing of faecal samples generated a metagenomic dataset of 258 million paired-end reads, with an average of 52 million paired reads per sample and >98% of reads from each sample surviving trimming (Table S1, available from https://doi.org/10.6084/m9.figshare.26968957).

**Fig. 1.**
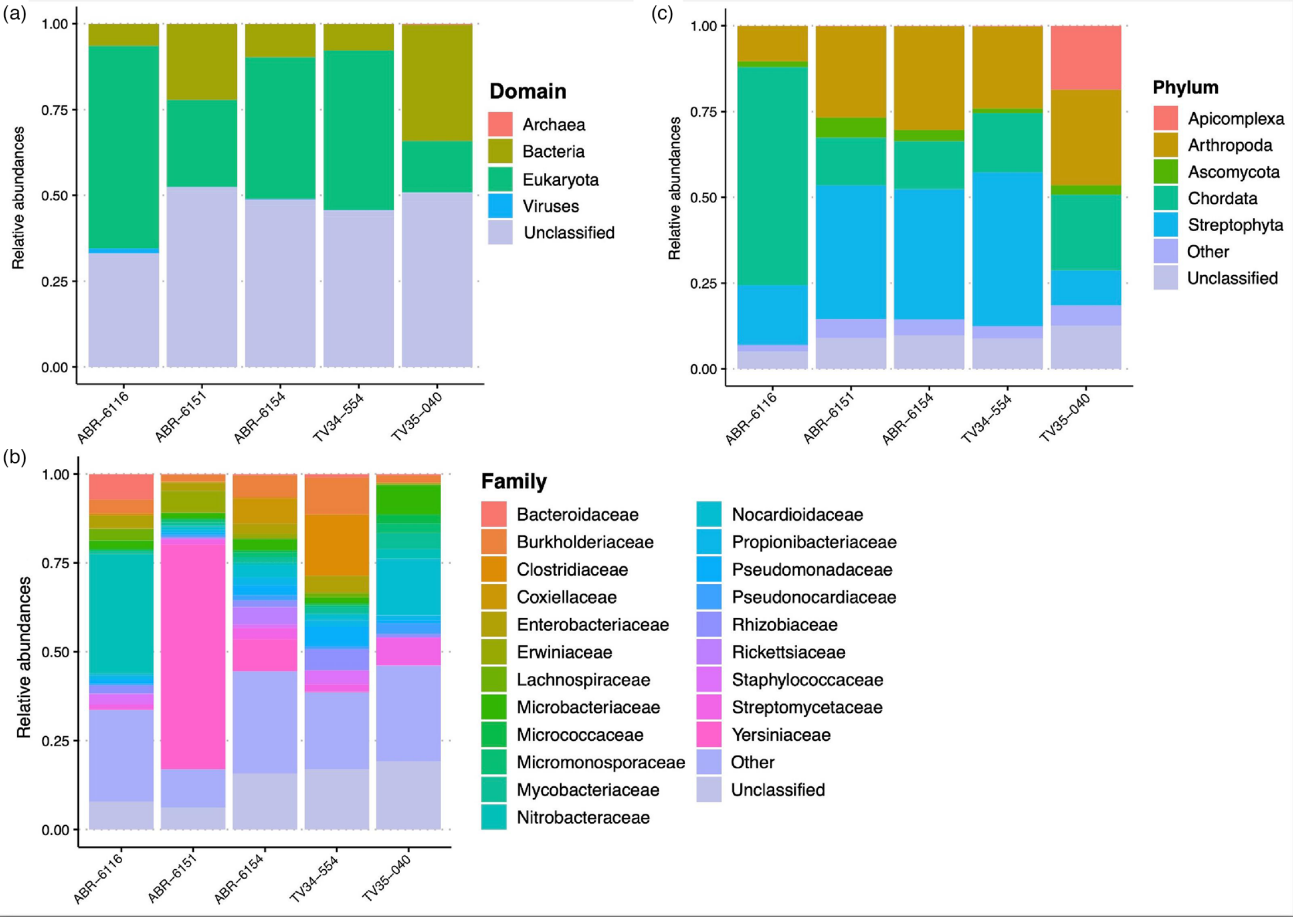
Read-based profiling of the parid faecal microbiota. (**a**) Relative abundance of the global community composition aggregated at the kingdom level. The read classification counts from Kraken2 were normalized using Total sum scaling normalization, and the counts were aggregated at the kingdom level. Counts classified as ‘root’ by Kraken2 were considered unclassified. (**b**) Relative abundance of the bacterial fraction. The counts were sub-setted for the bacterial fraction, transformed using TSS and aggregated at the family level. Families with a relative abundance below 2% in the samples were grouped as ‘other’ in this plot. (**c**) Relative abundance of the eukaryotic fraction. The counts were sub-setted for the eukaryotic fraction, transformed using TSS and aggregated at the phylum level. Phyla with a relative abundance below 2% in the samples were grouped as ‘other’ in this plot.

Taxonomic profiling of the reads using Kraken2 [41] revealed that 45–52% of the sequencing reads remained unclassified, confirming the largely unknown nature of the great tit and blue tit faecal microbiomes (Fig. 1 and Table S2). These analyses revealed substantial differences between samples in terms of ratios of reads assigned to host, food, gut microbiome and potential pathogens. Such variation – which we know from experience is not uncommon when analysing such microbiomes [5, 9, 38, 64, 65] – is likely to reflect differences in the shedding of host DNA from the gut, release of DNA from food components and recent dietary intake.

In ABR-6116, over a third of the reads were assigned to *Aves* (i.e. to the host), while in the other samples, this was <5 %. The relative abundance of reads assigned to phylum *Arthropoda* (home to most potential food animals, including spiders and insect larvae) was consistent across our samples, ranging from 4.17 to 12.5% of reads (Fig. 1). Great tits and blue tit nestlings are highly dependent on lepidopteran larvae (caterpillars) for a high-quality diet during development, but will provision young with lower quality invertebrates in urban environments, where caterpillar abundance is low [66, 67]. As the nests in our study were in high-quality oak woodland habitat, within a rural area, caterpillar abundance is expected to be high.

The phylum *Streptophyta* (potential food plants) accounted for ≥10% of reads in most samples, but for only 1.5% in great tit TV34-554. Two plant species featured a high relative abundance in the Kraken assignments. The sunflower *Helianthus annuus* accounted for 11.4% and 7.6% of reads from blue tits ABR6151 and ABR6154, while the oak *Quercus robur* accounted for 3.2% of reads in the remaining blue tit ABR6116 and for 3.9% in great tit TV35040. Although these results are generally consistent with a similar previous analysis using DNA metabarcoding [68], the presence of oak reads is best explained by the consumption of caterpillars that eat oak (e.g. oak tortrix moth caterpillars) rather than direct consumption of oak, while high abundance of sunflower reads suggests provisioning of garden bird seed by adults to their young. These findings highlight the wide-ranging human-influenced habitat of these wild birds.

Bacterial reads were predominantly assigned to the four phyla in the NCBI taxonomy most associated with animal gut microbiomes – *Proteobacteria*, *Firmicutes*, *Bacteroidetes* and *Actinobacteria* (Fig. 1), but also suggested the presence of potential pathogens. In one juvenile blue tit, ABR6154, a single bacterial species, *Serratia fonticola,* accounted for 8.8% of all sequence reads. Such monodominance of the gut microbiome by a single organism has been seen in other contexts, particularly in human neonates and adults who have received broad-spectrum antibiotics [5,69,71]. However, its significance in this context remains uncertain: given that the avian host appeared healthy, it might represent a normal step in the ecological succession required to build a gut microbiome in this species, or it might be associated with an inapparent decrease in fitness.

In the other juvenile blue tit, ABR6116, just over 1% of reads were assigned to a single viral species ‘Great tit siadenovirus A’. Siadenoviruses have been detected in various avian species, including great tits and blue tits, where they have been associated with renal pathology and mortality [72,73].

In the fledgling great tit TV34554, sequences assigned to the phylum *Apicomplexa* (obligate endoparasites of birds and other animals) accounted for 2.80% of reads. Most of these reads were assigned to the subclass *Coccidia* and to the family *Eimeriidae*. Importantly, it has been reported that the classification of morphologically defined genera among the eimeriid coccidia conflicts with sequence-based phylogenies [74].

These results, while providing a starting point for further investigations, may be confounded by a high prevalence of false positive detection. Indeed, read-based profiling is known to provide assignments at the level of genus and species that can be untrustworthy [75]. This is evident from our dataset in that many reads are quite implausibly assigned to bird species other than the great tit and blue tit. For example, in blue tit ABR6116, 11.4% of reads were assigned to the common reed warbler *Acrocephalus scirpaceus* and 7.2% to the European robin *Erithacus rubecula*. Taxonomic profiling also fails to provide whole-genome data or insights into functional diversity or population structure of the species they report. In addition, as this approach relies on a reference database, it can only report previously known organisms, which can limit the exploration of lesser-studied ecosystems. Given these limitations, we next explored the microbial communities associated with these birds through a sequence assembly approach.

### Recovery of mitochondrial genomes from faecal microbiome allows the genetic confirmation of the host species

Assemblies from the five faecal samples generated 504,238 contigs longer than 1,000 bp (Table S1). To confirm the identity of the host species, the great tit mitochondrial reference genome (GenBank ID: NC_040875.1) [76] was used as a query in high-stringency blastn searches of the metagenomic assemblies. This allowed us to retrieve 16 kb mitochondrial genomes from the great tit samples (available from https://doi.org/10.5281/zenodo.13734416) that both showed >99.9% sequence identity with the closest of the five available great tit mitochondrial genomes (GenBank ID: MN356395.1). We also identified three mitochondrial genomes from the blue tit samples, each of which showed 97% sequence identity to the only available complete *Cyanistes* mitochondrial genome, from the azure tit *Cyanistes cyanus* (GenBank ID: KX388472.1) [77], and >99.9% identity to *Cyanistes caeruleus* mitochondrial sequences from the control region and cytochrome b gene [78]. These sequences, which together share >99.9% nt identity, represent the first full mitochondrial genomes from *Cyanistes caeruleus* (available from https://doi.org/10.5281/zenodo.13734416).

Next, we sought to confirm the presence of apicomplexan parasites in the great tit samples. We used as a query in high-stringency tblastn searches of the metagenomic assemblies the cytochrome c oxidase subunit I protein sequence from *Isospora picoflavae* (GenBank ID: UQT67988). This species has been described from a woodpecker – the northern yellow*‐*shafted flicker *Colaptes auratus luteus* – but sits within a clade containing *Isospora* species from various passerine hosts [79]. This search allowed us to retrieve a single complete 6 kb *Isospora* mitochondrial genome from each of the great tit samples (available from https://doi.org/10.5281/zenodo.13734416). The *Isospora* mitochondrial genome retrieved from the great tit TV34-554 showed 99.4% nt identity to the mitochondrial genome from *Isospora* sp. JRBarta-2021c from the American robin, *Turdus migratorius* (GenBank ID MW645338;), 97.1% nt identity to the mitochondrial genome of *I. picoflavae* (GenBank: MW599989.1) and 97.7% nt identity to a partial cytochrome c oxidase subunit I sequence from a great tit in the Czech Republic (GenBank: MK573833.1) [80].

On the other hand, the *Isospora* mitochondrial genome from great tit TV35-040 showed 96.9% nt identity to the mitochondrial genome from *Isospora* sp. JRBarta-2021c from *T. migratorius*, 97.6% nt identity to the *I. picoflavae* mitochondrial genome and 95.4% nt identity to the partial cytochrome c oxidase from the great tit from the Czech Republic. This sequence had a 96.0% nt identity to the *Isospora* mitochondrial genome from great tit TV34-554.

Additionally, a blastn search of contigs with the small subunit ribosomal RNA gene from *I. picoflavae* (GenBank: MW618926.1) retrieved two sequences from great tit TV34-554 and TV35-040 that showed a 99% nt identity across the entire small subunit ribosomal RNA gene from *I. picoflavae* sequence (File S4).

Classically, *Isospora* species have been described based on the morphology of their sporulated oocysts, pathology and host identity, and no guidelines are available for the description of new species from sequence data alone [74,79]. Nonetheless, given the consistent results obtained with mitochondrial and rRNA sequences, it seems likely that the two great tits in our survey carry different species of *Isospora*, one shared with the American robin and the other apparently novel.

### MAGs document taxonomic and functional diversity

From the parid faecal samples, we generated 26 non-redundant bins from single-sample assemblies using three different approaches to binning (Table S3). A total of 18 bins represent medium- or high-quality MAGs. De-replication at 95% ANI clustered MAGs into 17 species clusters, spanning 7 phyla. Notably, four of these species have validly published Latin binomials, while one has been assigned alphanumerical placeholders by GTDB [81].

Among our MAG species clusters, 12 represent new candidate species within bacterial genera previously delineated by GTDB. Building on our recent efforts with the creation of arbitrary well-formed Latin names [82] and with the oesophageal microbiome [9], we have assigned *Candidatus* names (abbreviated as *Ca*.) to all the unnamed taxa revealed by our metagenomic analyses (Table 2).

**Table 2.** Protologues for newly named species. Protologues for new *species* identified by the analysis of MAGs from the faecal microbiomes of the great tit and blue tit

Species	Etymology	Description
*Ca*. Uzinuria cyanistis sp. nov.	*cyanistis. N.L. gen. masc n*. cyanistis, of *Cyanistes*, genus name of the host bird	A bacterial species identified by metagenomic analysis of a faecal sample from the Eurasian blue tit *Cyanistes caeruleus* and assigned to this genus according to the algorithms of the GTDB Toolkit operating on GTDB Release 220; this species includes all bacteria with genomes that show ≥95% ANI to the type genome for the species to which we have assigned the MAG ID ABR-6151_bin.14_sub and which is available via Zenodo archive 10.5281/zenodo.13643296. The G+C content in mol% of the type genome is 30%, and the genome length is 211,505 bp
*Ca*. Cedopifica cyanistis sp. nov.	*cyanistis. N.L. gen. masc n*. cyanistis, of *Cyanistes*, genus name of the host bird	A bacterial species identified by metagenomic analysis of a faecal sample from the Eurasian blue tit *Cyanistes caeruleus* and assigned to this genus according to the algorithms of the GTDB Toolkit operating on GTDB Release 220; this species includes all bacteria with genomes that show ≥95% ANI to the type genome for the species to which we have assigned the MAG ID ABR-6151_SemiBin_15 and which is available via Zenodo archive 10.5281/zenodo.13643296. The G+C content in mol% of the type genome is 42%, and the genome length is 1,442,697 bp
*Ca*. Cedopifica gen. nov.	*Cedopifica*. N.L. fem. n. Cedopifica, a name created arbitrarily, while preserving the phonotactics and morphology of Latin	A bacterial genus identified and delineated according to the algorithms of the GTDB Release 09-RS220; the GTDB alphanumeric placeholder designation for this genus is JAHHUI01; the type species for the genus is *Ca*. Cedopifica cyanistis; according to GTDB Release 09-RS220, this genus belongs to the family WRBN01
*Ca*. Cimetritia cyanistis sp. nov.	*cyanistis. N.L. gen. masc n*. cyanistis, of *Cyanistes*, genus name of the host bird	A bacterial species identified by metagenomic analysis of a faecal sample from the Eurasian blue tit *Cyanistes caeruleus* and assigned to this genus according to the algorithms of the GTDB Toolkit operating on GTDB Release 220; the GTDB alphanumeric placeholder designation for this genus is GCF-002259525; the arbitrary name *Ca*. Cimetritia has been published elsewhere [84]; this species includes all bacteria with genomes that show ≥95% ANI to the type genome for the species to which we have assigned the MAG ID ABR-6151_SemiBin_32 and which is available via Zenodo archive 10.5281/zenodo.13643296;the G+C content in mol% of the type genome is 33%, and the genome length is 1,507,853 bp
*Ca*. Udofia cyanistis sp. nov.	*cyanistis. N.L. gen. masc n*. cyanistis, of *Cyanistes*, genus name of the host bird	A bacterial species identified by metagenomic analysis of a faecal sample from the Eurasian blue tit *Cyanistes caeruleus* and assigned to this genus according to the algorithms of the GTDB Toolkit operating on GTDB Release 220; this species includes all bacteria with genomes that show ≥95% ANI to the type genome for the species to which we have assigned the MAG ID ABR-6151_SemiBin_38_sub and which is available via Zenodo archive 10.5281/zenodo.13643296; the G+C content in mol% of the type genome is 35%, and the genome length is 648,929 bp
*Ca*. Udofia gen. nov.	*Udofia,* N.L. fem. n. Udofia, a name created arbitrarily, while preserving the phonotactics and morphology of Latin	A bacterial genus identified and delineated according to the algorithms of the GTDB Release 09-RS220; the GTDB alphanumeric placeholder designation for this genus is CALTSX01; the type species for the genus is *Ca*. Udofia cyanistis; according to GTDB Release 09-RS220, this genus belongs to the family f__ CALTSX01
*Ca*. Pugiria cyanistis sp. nov.	*cyanistis. N.L. gen. masc n*. cyanistis, of *Cyanistes*, genus name of the host bird	A bacterial species identified by metagenomic analysis of a faecal sample from the Eurasian blue tit *Cyanistes caeruleus* and assigned to this genus according to the algorithms of the GTDB Toolkit operating on GTDB Release 220; this species includes all bacteria with genomes that show ≥95% ANI to the type genome for the species to which we have assigned the MAG ID ABR-6154_bin.9 and which is available via Zenodo archive 10.5281/zenodo.13643296; the G+C content in mol% of the type genome is 38%, and the genome length is 1,811,547 bp
*Ca*. Pugiria gen. nov.	*Pugiria,* N.L. fem. n. Pugiria, a name created arbitrarily, while preserving the phonotactics and morphology of Latin	A bacterial genus identified and delineated according to the algorithms of the GTDB Release 09-RS220; the GTDB alphanumeric placeholder designation for this genus is JAVHYG01; the type species for the genus is *Ca*. Pugiria cyanistis; according to GTDB Release 09-RS220, this genus belongs to the family *Diplorickettsiaceae*
*Ca*. Paridicola gen. nov.	*Paridicola*. N. L. gen. fem. n. parida, a bird from the family *Paridae*; L. masc./fem. n. suff. -cola, inhabitant of; N.L. masc./fem. n. paridicola, inhabitant of parids	A bacterial genus identified by metagenomic analyses; the genus includes all bacteria with genomes that show ≥60% average aa identity (AAI) to the genome of the type strain from the type species *Ca*. Paridicola cyanistisThis genus has been assigned by GTDB-Tk v1.3.0 working on GTDB Release 220 to the order *Legionellales* and to the family *Legionellaceae*
*Ca*. Paridicola cyanistis sp*.* nov.	*cyanistis. N.L. gen. masc n*. cyanistis, of *Cyanistes*, genus name of the host bird	A bacterial species identified by metagenomic analysis of a faecal sample from the Eurasian blue tit *Cyanistes caeruleus* and assigned to this genus according to the algorithms of the GTDB Toolkit operating on GTDB Release 220; this species includes all bacteria with genomes that show ≥95% ANI to the type genome for the species to which we have assigned the MAG ID ABR-6151_SemiBin_36 and which is available via Zenodo archive 10.5281/zenodo.13643296; the G+C content in mol% of the type genome is 40%, and the genome length is 2,370,451 bp
Ca. Rosutilega pari sp. nov.	*pari*. L. gen. masc. n. pari, of *Parus*, genus name of the host bird	A bacterial species identified by metagenomic analysis of a faecal sample from the great tit *P. major* and assigned to this genus according to the algorithms of the GTDB Toolkit operating on GTDB Release 220; this genus has been assigned by GTDB the alphanumeric designation JAVHYG01; the arbitrary name has been published elsewhere [84]; this species includes all bacteria with genomes that show ≥95% ANI to the type genome for the species to which we have assigned the MAG ID TV34-554_SemiBin_11 and which is available via Zenodo archive 10.5281/zenodo.13643296; the G+C content in mol% of the type genome is 68%, and the genome length is 1,776,244 bp
Ca. Methyloceanibacter pari sp. nov.	*pari*. L. gen. masc. n. pari, of *Parus*, genus name of the host bird	A bacterial species identified by metagenomic analysis of a faecal sample from the great tit *Parus major* and assigned to this genus according to the algorithms of the GTDB Toolkit operating on GTDB Release 220; this species includes all bacteria with genomes that show ≥95% ANI to the type genome for the species to which we have assigned the MAG ID TV34-554_SemiBin_4 and which is available via Zenodo archive 10.5281/zenodo.13643296; the G+C content in mol% of the type genome is 63%, and the genome length is 1,837,002 bp
Ca. Scandinavium pari sp. nov.	*pari*. L. gen. masc. n. pari, of *Parus*, genus name of the host bird	A bacterial species identified by metagenomic analysis of a faecal sample from the great tit *Parus major* and assigned to this genus according to the algorithms of the GTDB Toolkit operating on GTDB Release 220; this species includes all bacteria with genomes that show ≥95% ANI to the type genome for the species to which we have assigned the MAG ID TV35-040_maxbin.004_sub and which is available via Zenodo archive 10.5281/zenodo.13643296;the G+C content in mol% of the type genome is 50%, and the genome length is 2,647,412 bp
Ca. Mammaliicoccus pari sp. nov.	*pari*. L. gen. masc. n. pari, of *Parus*, genus name of the host bird	A bacterial species identified by metagenomic analysis of a faecal sample from the great tit *Parus major* and assigned to this genus according to the algorithms of the GTDB Toolkit operating on GTDB Release 220; this species includes all bacteria with genomes that show ≥95% ANI to the type genome for the species to which we have assigned the MAG ID TV35-040_maxbin.005_sub and which is available via Zenodo archive 10.5281/zenodo.13643296;the G+C content in mol% of the type genome is 34%, and the genome length is 8,553,045 bp
Ca. Agrobacterium pari sp. nov.	*pari*. L. gen. masc. n. pari, of *Parus*, genus name of the host bird	A bacterial species identified by metagenomic analysis of a faecal sample from the great tit *Parus major* and assigned to this genus according to the algorithms of the GTDB Toolkit operating on GTDB Release 220; this species includes all bacteria with genomes that show ≥95% ANI to the type genome for the species to which we have assigned the MAG ID TV35-040_SemiBin_28 and which is available via Zenodo archive 10.5281/zenodo.13643296;the G+C content in mol% of the type genome is 59%, and the genome length is 4,361,088 bp
Ca. Dwaynesavagella pari sp. nov.	*pari*. L. gen. masc. n. pari, of *Parus*, genus name of the host bird	A bacterial species identified by metagenomic analysis of a faecal sample from the great tit *Parus major* and assigned to this genus according to the algorithms of the GTDB Toolkit operating on GTDB Release 220; this species includes all bacteria with genomes that show ≥95% ANI to the type genome for the species to which we have assigned the MAG ID TV35-040_SemiBin_37 and which is available via Zenodo archive 10.5281/zenodo.13643296; the G+C content in mol% of the type genome is 30%, and the genome length is 1,518,404 bp

**Fig. 2.**
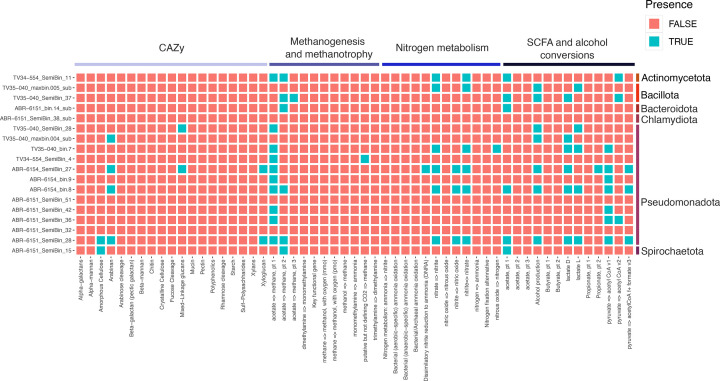
Predicted metabolic capability of the MAGs from the parid gut. Predicted coding sequences in each high- and medium-quality MAG were annotated using DRAM against the UniRef90, PFAM and dbCAN databases. The metabolic capacities of each MAG were inferred using the ‘distilled’ function from DRAM, and the presence/absence of metabolism of interest was plotted as a tile plot.

The metabolic capacity of each MAG was characterized using DRAM, predicting the high-level metabolic characteristics of these uncharacterized genomes (Fig. 2). Notably, MAGs representing the novel species *Cedopifica cyanistis* carried carbohydrate-active enzymes involved in the cleavage of amorphous cellulose (GH5), suggesting a potential role of this species in the passerine digestive function.

Consistent with the read-based profiling results that showed the presence of *Serratia fonticola* at high relative abundance in the blue tit ABR-6154, we assembled a high-quality MAG for this species. *Serratia fonticola* is a recognized pathogen of humans associated with gastrointestinal, respiratory, urinary, bloodstream, soft tissue and joint infections [83] and has been isolated from a range of environmental sources, including wild and domesticated birds. However, it has not previously been seen in wild parids [84,86].

### Antiobiotic resistance genes in the parid gut

Next, we surveyed the repertoire of antibiotic and metal resistance genes found in the passerine faecal microbiota. Interestingly, we identified a large repertoire of antibiotic resistance genes, covering genes potentially involved in the resistance to beta-lactams, tetracycline, fluoroquinolones and polymyxins. Notably, 43 (17 %) of these resistance genes were detected on a putative plasmid and so are potentially mobile (File S4). Resistanc0e genes were associated with a variety of bacterial hosts, in particular the genera *Rahnella*, *Pseudomonas* and *Serratia*.

Resistance to beta-lactams in *Serratia fonticola* is mediated by chromosomal class A extended-spectrum beta-lactamases belonging to the FONA family, encoded by the *blaFONA* genes [85], and we confirmed the presence of this gene in the gut microbiome of blue tit ABR-6154, carried on a contig sequence classified as *Serratia fonticola*. Concerns have been raised that wild birds can harbour clinically significant resistance genes and could potentially play a role in the global dissemination of antibacterial resistance [87,91]. However, whether the presence of this antimicrobial-resistant pathogen in wild birds in the UK represents a risk to human health remains unclear without an in-depth analysis of the population structure of *Serratia fonticola*.

### A diverse community of phages and eukaryotic viruses

**Fig. 3.**
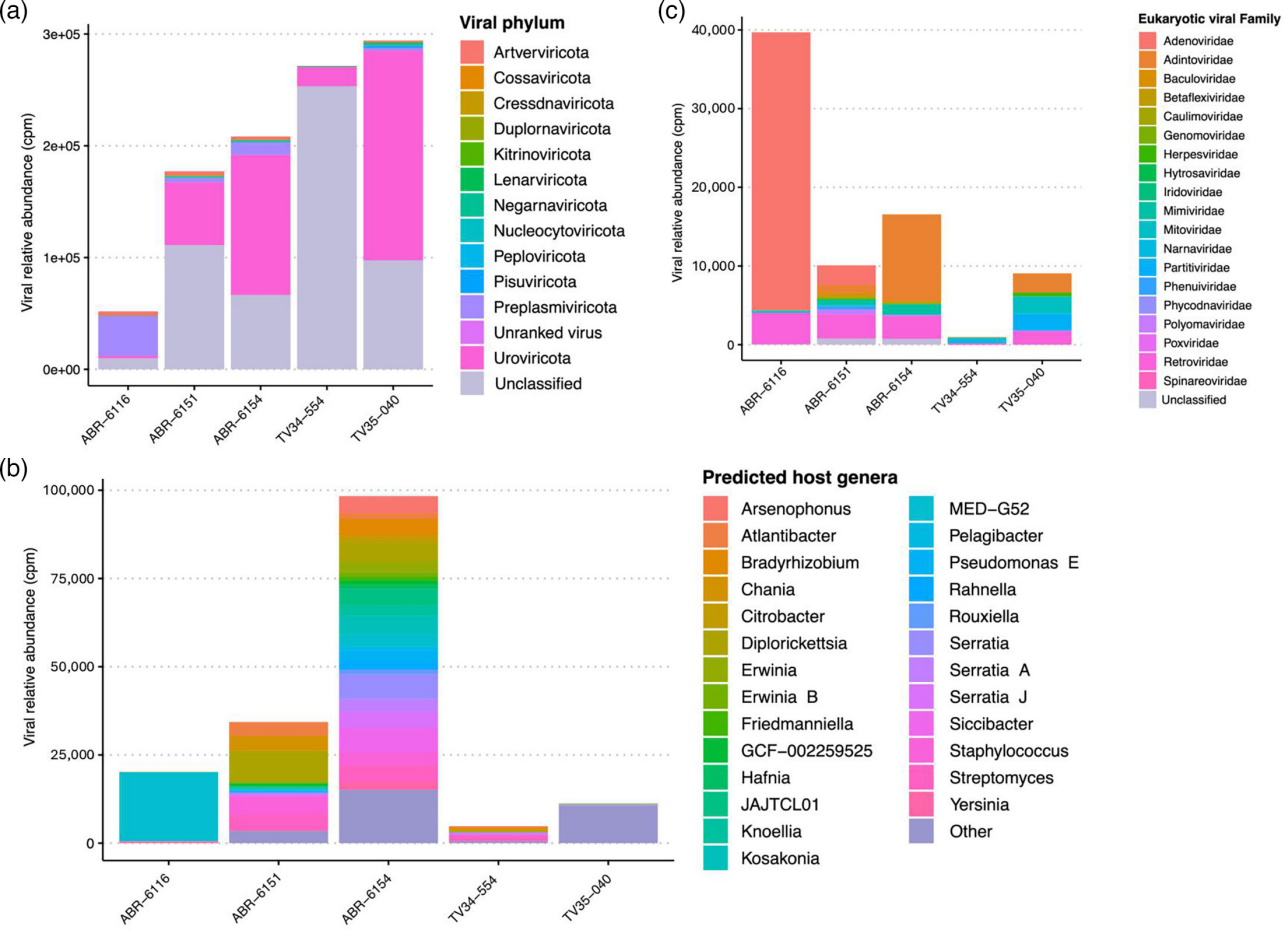
Overview of the parid faecal virome. (**a**) Relative abundance of the global viral community composition aggregated at the phylum level. The reads were mapped against the non-redundant vOTU catalogue, and the counts were normalized as read count per kb and million reads (cpm) before being aggregated at the phylum level. (**b**) Predicted bacterial hosts of the bacteriophage community. The viral community read counts were sub-setted for the Caudovirales fraction with a putative bacterial host identified. The cpm counts were aggregated at the host genus level, and the genera accounting for less than 200 cpm in the samples were grouped as ‘other’. (**c**) Relative abundance of the eukaryotic viral fraction. The read counts were sub-setted for the eukaryotic viral phylum and aggregated at the viral family level.

Finally, we explored viral diversity in these samples. A total of 1,870 viral sequences longer than 1 kb were identified, corresponding to 1,843 species-level vOTUs. We assessed the relative abundance of these viral species in the samples by read mapping against the representative genome of each vOTU, showing a very varied viral load and global composition between the samples (Fig. 3). The phage community was dominated by viruses from the phylum *Caudovirales* and accounted for 5–63 % of the viral content of the samples. Putative bacterial hosts were predicted for 360 Caudovirales vOTUs, with a close correspondence between predicted bacterial hosts and the main bacterial taxa identified in the samples (Fig. 3).

We identified 173 sequences classified as eukaryotic viruses, from a wide variety of viral families (Fig. 3). As expected from read-based profiling, the viral community in the juvenile blue tit ABR6116 was dominated by two Adenovirus vOTUs [73]. One of these, represented by the 26 kbp contig MEGAHIT-ABR-6151_935 (available from https://doi.org/10.5281/zenodo.13643307), showed 99.16% nt sequence identity with the complete genome of great tit adenovirus 3 (GenBank ID: MW508338.2), derived from a great tit found dead in Germany. This high degree of sequence identity, which includes 99% identity at the aa level for the viral DNA polymerase, indicates that this blue tit siadenovirus belongs to the species *Siadenovirus carbocapituli*, which so far has been found only in great tits [73].

The juvenile blue tit ABR6154 had a high relative abundance of five vOTUs belonging to the Adintoviridae family, a viral family identified in several wild animals and in humans, but not previously reported in wild birds [92].

## Conclusions

Compared to the guts of humans and domesticated animals, the microbiology of the wild bird gut remains largely unexplored. Here, we deliver new insights into this important ecosystem while also showcasing the advantages of shotgun metagenomics in providing catalogues of genes and genome sequences that take us well beyond what can be achieved using 16S ribosomal RNA gene sequences. Exploration of just five faecal samples allowed the recovery of gene and genome sequences from viruses, coccidian parasites and new bacterial species, substantially increasing the known microbial diversity of these habitats. In addition, we have been able to recover mitochondrial genomes from the host species, including the first mitochondrial genomes from the Eurasian blue tit. Deposition of such sequences into publicly available databases will strengthen all future studies, improving the quality of reference-based taxonomic assignments.

Although our focus here is on microbial ecology, phylogenetic profiling of metagenomic reads has provided informative but incomplete insights into host diet, paving the way for the delivery of more definitive insights using sophisticated bioinformatic approaches twinned with extensive genome sequencing of the UK flora and fauna [93,94]. Similarly, attempts to quantify parasite burden from metagenome sequences are likely to benefit from previous efforts using DNA barcodes [95].

Given the small number of samples analysed in this pilot study, we cannot hope to provide a comprehensive view of taxonomic diversity within the great tit and blue tit gut microbiomes. Nonetheless, this work gives us a tantalizing glimpse of the richness that awaits us when such approaches are fully optimized and rolled out more widely and established shotgun metagenomics as a potentially important tool that could help humans help birds address the challenges of the Anthropocene.

## Abbreviations

ANI: average nt identity
ARG: antibiotic resistance genes
GTDB: Genome Taxonomy Database
MAG: metagenome-assembled genome
vOTU: viral operational taxonomic unit
